# Uncovering key molecular mechanisms in the early and late-stage of papillary thyroid carcinoma using association rule mining algorithm

**DOI:** 10.1371/journal.pone.0293335

**Published:** 2023-11-02

**Authors:** Seyed Mahdi Hosseiniyan Khatibi, Sepideh Zununi Vahed, Hamed Homaei Rad, Manijeh Emdadi, Zahra Akbarpour, Mohammad Teshnehlab, Saeed Pirmoradi, Effat Alizadeh

**Affiliations:** 1 Clinical Research Development Unit of Tabriz Valiasr Hospital, Tabriz University of Medical Sciences, Tabriz, Iran; 2 Kidney Research Center, Tabriz University of Medical Sciences, Tabriz, Iran; 3 Rahat Breath and Sleep Research Center, Tabriz University of Medical Science, Tabriz, Iran; 4 Department of Computer Engineering, Abadan Branch, Islamic Azad University, Abadan, Iran; 5 Department of Electric and Computer Engineering, K.N. Toosi University of Technology, Tehran, Iran; 6 Drug Applied Research Center, Tabriz University of Medical Sciences, Tabriz, Iran; 7 Faculty of Advanced Medical Sciences, Department of Medical Biotechnology, Tabriz University of Medical Sciences, Tabriz, Iran; The First Hospital of Jilin University, CHINA

## Abstract

**Objective:**

Thyroid Cancer (TC) is the most frequent endocrine malignancy neoplasm. It is the sixth cause of cancer in women worldwide. The treatment process could be expedited by identifying the controlling molecular mechanisms at the early and late stages, which can contribute to the acceleration of treatment schemes and the improvement of patient survival outcomes. In this work, we study the significant mRNAs through Machine Learning Algorithms in both the early and late stages of Papillary Thyroid Cancer (PTC).

**Method:**

During the course of our study, we investigated various methods and techniques to obtain suitable results. The sequence of procedures we followed included organizing data, using nested cross-validation, data cleaning, and normalization at the initial stage. Next, to apply feature selection, a t-test and binary Non-Dominated Sorting Genetic Algorithm II (NSGAII) were chosen to be employed. Later on, during the analysis stage, the discriminative power of the selected features was evaluated using machine learning and deep learning algorithms. Finally, we considered the selected features and utilized Association Rule Mining algorithm to identify the most important ones for improving the decoding of dominant molecular mechanisms in PTC through its early and late stages.

**Result:**

The SVM classifier was able to distinguish between early and late-stage categories with an accuracy of 83.5% and an AUC of 0.78 based on the identified mRNAs. The most significant genes associated with the early and late stages of PTC were identified as (e.g., ZNF518B, DTD2, CCAR1) and (e.g., lnc-DNAJB6-7:7, RP11-484D2.3, MSL3P1), respectively.

**Conclusion:**

Current study reveals a clear picture of the potential candidate genes that could play a major role not only in the early stage, but also throughout the late one. Hence, the findings could be of help to identify therapeutic targets for more effective PTC drug developments.

## Introduction

Thyroid cancer is the most prevalent type of cancer within the category of endocrine malignancies [1]. Furthermore, it counts as the sixth most common cancer in women due to the increasing incidence rate [2]. Radiation exposure and environmental stimuli are the risk factors for the incidence of TC [3]. Thyroid cancer contains four histopathological subtypes, including Papillary Thyroid Cancer (PTC), Follicular Thyroid Carcinoma (FTC), Medullary, and Anaplastic [4]. Moreover, PTC and FTC are subordinates of Differentiated Thyroid Cancer (DTC), which together account for the majority of thyroid malignancies. PTC contains 75–80% of TC cases, and other subtypes occur less frequently. Based on the American Cancer Society report, the survival rate of PTC is about 100% in the early stage (stage I and stage II), while it decreases to 55% in the late stage (stage III and stage IV) diagnosis [5]. Thus, early detection of PTC through the use of novel methods and biomarkers is crucial. In addition, determining the key components of the genomic profiles can help figure out the Dominant Molecular Mechanisms in both stages of PTC.

Fine Needle Aspiration (FNA) biopsy and cytological categorization is the diagnostics reference method in TC. However, the accuracy of FNA depends on operator skill, an intrinsic characteristic of nodules, and cytology interpretation [6]. Due to these limitations of FNA cytology, several studies have been conducted to identify biomarkers for TC diagnosis in recent years. For instance, HBME-1 with CK19 combination [7] and LGALS3 [8] have been reported as diagnostic biomarkers. According to some researchers’ studies, the severity of TC is associated with EGFR [9]. Besides, several studies have tried to understand the relation of genomic features with survival and progression [10–13]. For example, Chai et al. have shown the association of BRAF high expression with tumor aggressiveness [12]. Another study has done benign and malignant TC classification based on RNA expression and reported 84% specificity on the validation data [14]. Researchers have reported varying expressions in the transforming growth factor, CDH1, COL1A1, CTNNA1, ITGA3, and FN1 in benign and malignant nodules of TC [15]. The significant association of TC subtypes and TC early detection with methylation data, such as RASSF1, DAPK1, and ESR1, has also been pointed in the literature [16]. Moreover, high expression of VDR is related to some phenotypes, including tall cell subtype, stage IV, and low recurrence-free survival of TC [10].

These works mainly studied the pathogenesis and progression of TC to find meaningful genomic features. They can help clinicians understand the pathogenesis of TC clearly. However, there is a necessity to find significant genomic features that can detect PTC at an early stage. Therefore, the present study aims to decode the molecular mechanism of the early/late stage of PTC as the first phase, and the stage prediction of PTC patients based on RNA-seq data, in the second phase. There is no doubt that PTC diagnosis in its early stages can help doctors to select more effective treatments, along with better monitoring of the patients. Additionally, decoding molecular mechanisms at the starting points can facilitate the development of new treatment schemas for PTC patients.

Nowadays, researchers apply various machine-learning algorithms to decipher molecular mechanisms of different diseases [17,18]. As a means to understand the importance of mRNA transcripts and categorizing the early or late stage of the patients, we have employed machine learning methods. To begin with, pre-processing phase including data organization, nested cross-validation, data cleaning, and normalization have been put to use. Secondly, statistical t-test and binary Non-Dominated Sorting Genetic Algorithm II (NSGAII) were applied in the feature-selection step, as filter and wrapper approaches, respectively. Subsequently, machine learning and deep learning classifiers were utilized to evaluate the selected features (mRNAs). Finally, the association rule mining algorithm was applied to the selected features to determine the significant mRNAs, as a means to help decoding of the dominant molecular mechanisms in the early and late stages of PTC.

## Material and method

### Data

The NCI’s GDC (Genomic Data Commons) data portal (https://portal.gdc.cancer.gov/) provides the RNA-seq and mRNA-seq data from TCGA (The Cancer Genome Atlas) repository. At the time of the study, the dataset contained 60,483 mRNA-seq transcripts for 554 PTC patients measured using the HTSeq-FPKM technique. In addition, clinical data of PTC patients were obtained from the cBioPortal database. We divided PTC patients into two groups based on cancer stage, including early-stage (stage I and II) with 375 samples and late-stage (III and IV) with 179 patients. This study was conducted according to the principles of the Declaration of Helsinki (2013). We received permission to access the research data file in the TCGA program from the National Cancer Institute, US. Approval was waived by the local ethics committee, as TCGA data are publicly available. Also, the local ethics code is: IR.TBZMED.REC.1400.065.

A second dataset namely GSE 60542 [19] was utilized to verify the results obtained from the TCGA dataset. GSE 60542 employed Affymetrix Human Genome U133 Plus 2.0 Array, the GPL 570 platform, to report the expression of 54675 mRNAs for each sample. The dataset comprises a total of 61 PTC patients, out of which 33 were early-stage and 38 were late-stage patients.

### Method

**Fig 1:**
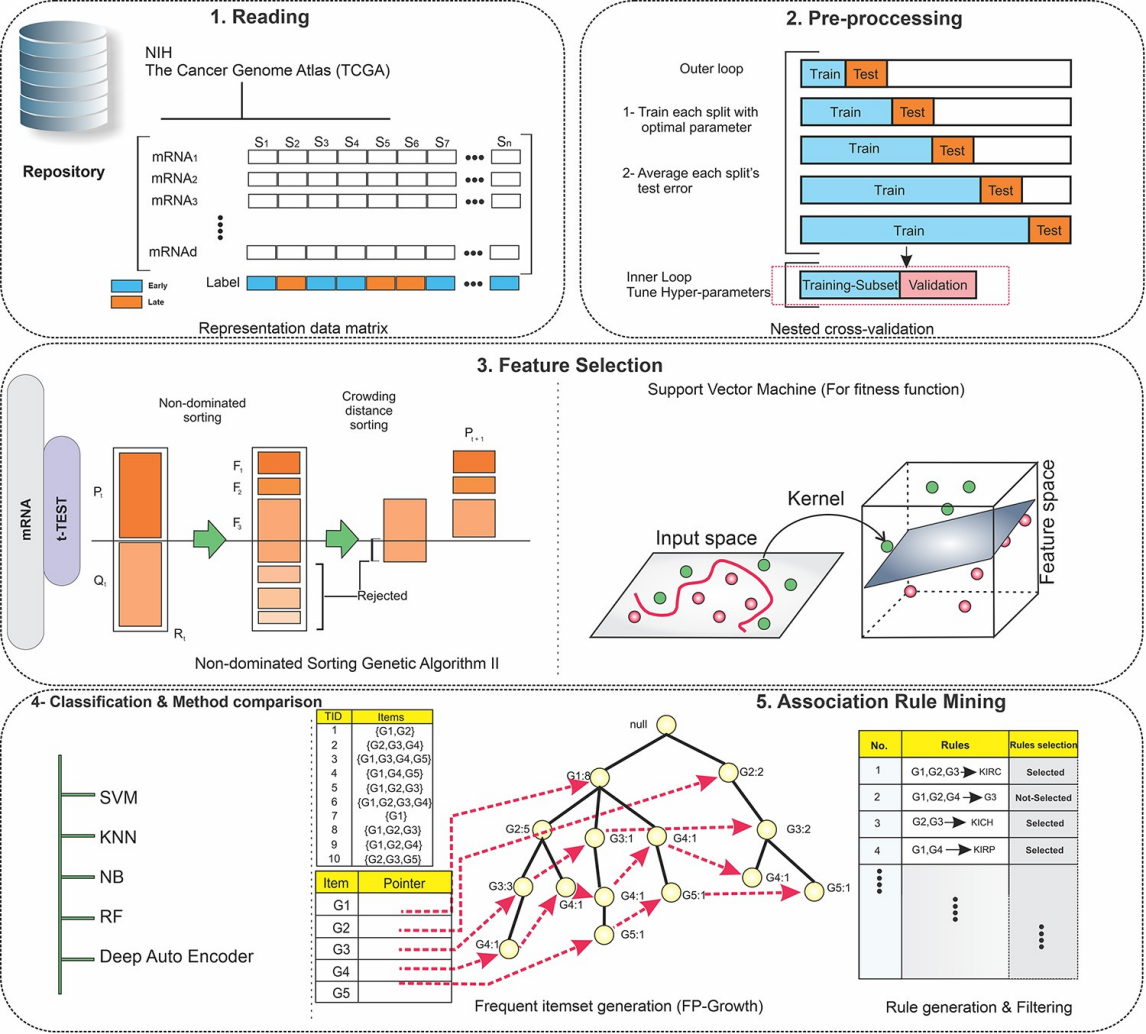
The overview of the proposed method. Five main steps, including reading, preprocessing, feature selection, classification, and association rule mining were applied to the mRNA expression data. 1) Required data was collected from the TCGA repository and got organized during the reading step. 2) The pre-processing step includes two sub-steps, nested cross-validation and data normalization. 3) The feature-selection step contains two parts: the filter method based on a t-test and the wrapper method based on binary Non-Dominated Sorting Genetic Algorithm II (NSGAII) for mRNA data, in which candidate mRNAs with more relevance to early-stage and late-stage Papillary Thyroid Cancer (PTC) were selected. 4) Multi-classifier models were utilized to evaluate the discrimination power of the selected mRNAs. 5) The Association Rule Mining method was employed to discover the possible hidden relationship between the selected mRNAs and early and late stages of PTC firstly, and the complex relationship among the selected mRNAs secondly.

The proposed approach for finding significant mRNAs has five steps, including reading, pre-processing, feature selection, classification, and application of association rule mining. Fig 1 illustrates the entire process and discloses more detail on each step.

During the reading step, the mRNA data was organized into a matrix of 554 rows and 60,483 columns, representing the number of samples and features, respectively. Next, the nested Cross-Validation (CV) approach was used to separate several Test proportions of data to achieve a more authentic error estimation, close to real-world situations. The number of folds was set to 10 for the outer loop and 5 for the inner loop in the nested CV. Meanwhile, some feature columns were removed, which had identical values for all samples of training folds of the inner loops. Last but not least, z-score and min-max methods were employed to normalize the feature-selection/classification and association rule mining steps, respectively.

The two-part feature-selection step helped to lower the number of features (mRNAs) by omitting the irrelevant attributes. The initial selection procedure was a classifier-independent filter method in which features were evaluated individually. T-test was used during this phase to reduce the dimension of mRNA data and it also contributed to alleviation of computational cost during the next wrapper step. We applied T-test to training folds of each inner loop and selected 15 top features based on their p-values subsequently. This process was repeated 50 times (10-fold in the outer loop and 5-fold in the inner loop). At the end of the procedure, 255 mRNAs were picked out considering the union of selected features obtained from training folds of inner-outer loops.

The succeeding wrapper method that we used for feature selection was the binary Non-Dominated Sorting Genetic Algorithm II (NSGAII). It is a classifier-dependent search method inspired by natural selection and we employed Support Vector Machine (SVM) for the fitness function evaluation. For robustness and reliability reasons, we defined the fitness function based on AUC, shown in Eq 1. Moreover, fitness function values, including mean and standard deviation of AUC, were calculated based on inner validation folds of each outer fold. Ultimately, 60 significant mRNAs were selected considering the output of binary NSGAII. The parameters for the algorithm including the number of population and number of iterations, were set to 40 and 50, respectively.


fitnessvalue=(1−mean(AUCinallvalidationfolds)+mean(standarddeviationofAUCinallvalidationfolds) (1)

To assess the differentiation power of the selected features (mRNAs), we utilized several supervised classifiers including SVM, Naive Bayes (NB), K-Nearest Neighbor (KNN), Random Forest (RF), and Deep Self-Organizing Auto-Encoder (SOAE) [20]. Key evaluation metrics such as accuracy, AUC, F1-score, sensitivity, and specificity were also calculated for each classifier.

In the next step, we studied significant relationships using association rule mining algorithm. We extracted association rules in regard to the relationship between selected mRNAs and early/late PTC stage groups. Here is how the process was carried out: The early/late-stage group was added as a new feature to mRNA data. The mRNAs were then categorized into three parts based on their expression levels: low, medium, and high. Next, the FP-Growth algorithm was used to generate association rules in two phases: frequent itemsets and rules generation. Early and late stages association rules were then extracted based on the consequent part of the rules and their values. To finalize rule analysis, we studied the antecedent part of the early-stage association rules and reported mRNAs, based on their repeat count in early-stage and late-stage rule sets. In the end, we considered the three top mRNAs of the early-stage and late-stage for a more in-depth investigation from a biological point of view, according to their repeat count. The parameters for the association rule mining algorithm, including min-support (frequent itemset), max-length (maximum length of frequent itemset), and lift (association rule), were set to 0.3, 4, and 1.1, respectively.

The further technical explanation for each of the stages mentioned in the methodology is covered comprehensively in the Supplementary part. In addition, the output results of each step are reported in the results section of the study. It is also worth mentioning that Python was the main programming language of choice in this study and several libraries and frameworks, namely Numpy, Pandas, Matplotlib, Scikit-learn, Scipy, PyTorch, Pymoo, and Mlxtend were employed to implement the proposed steps.

### Non-Dominated Sorting Genetic Algorithm II (NSGAII)

Multi-objective optimization is an important factor in the multi-criteria decision-making process. It has been employed in many research areas, including engineering, economics, and medicine, in which finding the optimal solution is needed for multi-objective difficulties. Trade-offs between two or more conflicting objectives are the fundamental concept in multi-objective problems, which various algorithms have been designed to solve. Meta-heuristic algorithms are robust procedures in optimization problems with incomplete data or limited calculation ability. Meta-heuristics do not find a globally optimal solution to some problems versus other optimization algorithms and iterative methods [21].

NSGA-II is the well-known meta-heuristic optimization method for multi-objective optimization [22]. Its full name is "Non-dominated Sorting Genetic Algorithm II" and is the updated version of NSGA. It finds solutions for non-convex, non-smooth single and multi-objective optimization problems. It utilizes a new mating mechanism based on the crowding distance and considering constraints using an adapted explanation of dominance, without applying penalty functions. The whole process of NSGAII algorithm is illustrated in Table 1.

**Table 1:** Pseudocode of NSGAII [23].

**Algorithm:** Non-dominated Sorting Genetic Algorithm (NSGA-II)
1. Create an initial population P_0_ 2. Create an offspring population Q_0_ 3. t = 0 4. **while** stopping criteria are not reached **do:** 5. R_t_ = P_t_ ∪ Q_t_ 6. F = fast-non-dominated-sort (R_t_) 7. P_t+1_ = ∅ and i = 1 8. **while** |P_t+1_| + |F_i_ | ≤ N **do:** 9. Apply crowding-distance-assignment (F_i_) 10. P_t+1_ = P_t+1_ ∪ F_i_ 11. i = i + 1 ** end** 12. Sort (F_i_, ≺ n) 13. P_t+1_ = P_t+1_ ∪ F_i_ [N − |P_t+1_|] 14. Q_t+1_ = create_new_pop (P_t+1_) 15. t = t + 1 **end**

### Association rule mining

Association rule mining is a powerful data mining tool that presents the hidden association in the form of rules by discovering associated frequently co-occur items in the dataset. Market basket analysis [24] and bioinformatics [25] are two main areas that apply association rule mining to extract the significant association in marketing and genomic data, respectively. The interpretation of gene expression data (mRNA), annotations, detection of protein interactions, and biomolecular localization predictions are some applications of the association rule mining in bioinformatics [25].

Association rule mining has two main steps, frequent itemset mining, and association rule generation. Frequent itemset mining (FIM) extracts frequently co-occur sets of items (i.e., frequent itemsets). If itemset support value is more than the minimum support threshold, itemset is called a frequent itemset. Next, the association rule generation step creates the rules from the discovered Frequent Itemsets (FIs). Next, the association rule generation step creates the rules from the discovered Frequent Itemsets (FIs). If support/confidence/lift value of the rule is no less than the minimum support/confidence/lift threshold, the generated rule is called the association rule. These thresholds are user-defined parameters.

The association rule mining is an NP-hard problem, in which finding the results are challenging in a reasonable time. Introducing the Apriori algorithm addressed the computational problem in most regular-sized data [26]. Since then, many types of research have been done to develop new algorithms such as FP-Growth [27] and Eclat [28]. These algorithms improved the scalability of the Apriori algorithm. However, the relative computational cost of the FIM stage during association rule mining for high-dimensional data and big data is still a challenging issue.

The above section mentions some principal ideas and terms used in association rule mining. We have described these ideas in an extended formulized fashion in supplementary part. Besides, the related theories are available in [29] with more details. Association rule mining has also been put to use in our recent work as well [30].

## Results

We have set two primary objectives for the current study. First, to identify significant mRNAs that can help classify early-stage and late-stage PTC groups with the best performance. Second, to decode the molecular mechanisms involved in those stages and single out the top mRNAs with the most associative interactions.

Table 2. reports the 60 most significant features among 60,483 mRNAs, which have been selected after two consecutive feature selection steps, using a t-test (filter method) and binary NSGAII algorithm (wrapper method).

**Table 2:** List of selected mRNAs in the feature selection step.

No.	mRNA ID	No.	mRNA ID	No.	mRNA ID
1	ENSG00000044574.7	21	ENSG00000173933.18	41	ENSG00000234709.2
2	ENSG00000060339.12	22	ENSG00000175110.10	42	ENSG00000236548.1
3	ENSG00000111886.10	23	ENSG00000178163.6	43	ENSG00000241294.1
4	ENSG00000113739.9	24	ENSG00000181171.5	44	ENSG00000249077.1
5	ENSG00000128594.6	25	ENSG00000184617.10	45	ENSG00000253120.1
6	ENSG00000129480.11	26	ENSG00000184678.9	46	ENSG00000254463.1
7	ENSG00000137500.8	27	ENSG00000185274.10	47	ENSG00000256196.1
8	ENSG00000143603.17	28	ENSG00000196787.3	48	ENSG00000256590.2
9	ENSG00000144057.14	29	ENSG00000211648.2	49	ENSG00000258449.1
10	ENSG00000145248.6	30	ENSG00000221553.1	50	ENSG00000258813.2
11	ENSG00000150433.8	31	ENSG00000224104.1	51	ENSG00000260498.4
12	ENSG00000151883.15	32	ENSG00000224287.2	52	ENSG00000261403.1
13	ENSG00000153822.12	33	ENSG00000224616.1	53	ENSG00000262038.1
14	ENSG00000154144.11	34	ENSG00000225470.5	54	ENSG00000265337.1
15	ENSG00000154217.13	35	ENSG00000227741.1	55	ENSG00000269043.1
16	ENSG00000158373.8	36	ENSG00000228800.1	56	ENSG00000270822.1
17	ENSG00000159200.16	37	ENSG00000231475.3	57	ENSG00000272963.1
18	ENSG00000163686.12	38	ENSG00000233191.1	58	ENSG00000274698.1
19	ENSG00000170011.12	39	ENSG00000233235.1	59	ENSG00000277965.1
20	ENSG00000171208.8	40	ENSG00000233822.4	60	ENSG00000280660.1

For evaluating the chosen features (mRNAs) in regards to their differentiation power of early and late stages, five classifiers including SVM, KNN, NB, RF, and deep Self-Organizing Auto-Encoder were employed. Table 3 demonstrates the average performance of each classifier by reporting primary evaluation metrics such as accuracy, F1-score, AUC, sensitivity (Sn), and specificity (Sp) for train/validation/test folds of the candidate mRNAs.

**Table 3:** The performance of classifiers based on 60 selected mRNAs.

Classifier	Folds	Accuracy (%)	AUC-ROC	F1-score (Early stage)	F1-score (Late stage)	MCC	Sn	Sp
SVM	Train	89.2	0.86	0.92	0.82	0.75	0.77	0.95
	Validation	81.5	0.76	0.86	0.67	0.56	0.6	0.91
	Test	**83.5**	**0.78**	**0.88**	**0.71**	**0.61**	0.66	0.91
KNN	Train	81.8	0.8	0.89	0.73	0.59	0.77	0.81
	Validation	72.8	0.72	0.78	0.62	0.42	0.7	0.74
	Test	73	0.73	0.78	0.64	0.45	**0.75**	0.71
NB	Train	71.5	0.75	0.75	0.66	0.47	0.85	0.64
	Validation	69	0.71	0.73	0.62	0.4	0.79	0.63
	Test	71.1	0.71	0.76	0.59	0.41	0.73	0.7
RF	Train	82	0.73	0.88	0.63	0.58	0.47	0.98
	Validation	73.6	0.62	0.82	0.42	0.34	0.3	0.94
	Test	75.4	0.65	0.81	0.49	0.4	0.39	**0.92**
AE	Train	79	0.73	0.85	0.62	0.5	0.54	0.91
	Validation	77	0.7	0.83	0.51	0.44	0.51	0.89
	Test	78.4	0.73	0.84	0.62	0.48	0.57	0.88

The performance of classifiers based on mRNA features shows that SVM tops other algorithms with an Accuracy of 83.5 and an AUC-ROC score of 0.78 in Test data evaluation.

We also considered another dataset (GSE 60542) for evaluating the selected features. Table 4 shows the performance of the SVM classifier with the same settings for the evaluation dataset. The attempt was to select 60 mRNAs from a pool of 54675 mRNAs using their related gene names. However, we could only find 30 common mRNAs between the two distinct datasets, which is essentially due to the different platforms used to measure mRNA expression. Therefore, only the common features were taken into consideration and the remaining 30 mRNAs in the feature vector were nullified having zero value, being fed into the SVM classifier.

**Table 4:** The performance of the SVM classifier based on 60 (30 plus 30-zeroed) selected mRNAs for GSE 60542 dataset.

Classifier	Folds	Accuracy (%)	AUC-ROC	F1-score (Early stage)	F1-score (Late stage)	MCC	Sn	Sp
SVM	Train	100	1	1	1	1	1	1
	Test	77	0.76	0.81	0.72	0.53	0.69	0.83

The performance of the proposed method also stands out better compared to recent study on the same data with a reported 71.57/75.49 accuracy and 0.66/0.72 AUC in various subsets of mRNA features [5].

In addition to achieving improved metrics, we discovered interesting relations including feature(s)-feature(s) (mRNAs) and feature(s)-target (early-stage/late-stage) during the association rule mining step. Furthermore, we selected the most associative significant mRNAs based on the repeat count of these features in generated rules and studied their role in the early and late stages of PTC tumors.

### mRNA data association rule mining analysis

14 top mRNAs are shown in Table 5, which have been extracted considering their repeat count in association rules. Among the early-stage association rules, ENSG000001781163.6, ENSG00000129480.11, and ENSG00000060339.12 were the top ones with 28, 15, and 13 repeat counts, respectively. On the other hand, ENSG00000233191.1, ENSG00000254463.1, and ENSG00000224287.2 were the top features with 463, 104, and 94 repeat counts in the group of late-stage associations. In addition, the top twenty mined association rules for the early-stage and late-stage are illustrated separately in the form of “if-then rules” throughout Table 6.

**Table 5:** Top mRNAs based on repeat count in early-stage and late-stage rules.

No.	Early-stage rules		Late-stage rules	
	mRNA ID	Repeat Count	mRNA ID	Repeat Count
1	**ENSG00000178163.6**	**28**	**ENSG00000233191.1**	**463**
2	**ENSG00000129480.11**	**15**	**ENSG00000254463.1**	**104**
3	**ENSG00000060339.12**	**13**	**ENSG00000224287.2**	**94**
4	ENSG00000170011.12	2	ENSG00000184617.10	82
5	ENSG00000144057.14	2	ENSG00000272963.1	81
6	ENSG00000280660.1	2	ENSG00000233235.1	78
7	ENSG00000269043.1	2	ENSG00000159200.16	74
8	ENSG00000256590.2	2	ENSG00000260498.4	72
9	ENSG00000256196.1	2	ENSG00000277965.1	72
10	ENSG00000253120.1	2	ENSG00000113739.9	68
11	ENSG00000233822.4	2	ENSG00000154217.13	65
12	ENSG00000231475.3	2	ENSG00000261403.1	61
13	ENSG00000228800.1	2	ENSG00000262038.1	59
14	ENSG00000181171.5	2	ENSG00000224104.1	58

**Table 6:** Twenty of the top early-stage and late-stage rules based on sorted lift value.

**Twenty of the top early-stage rules**								
**No.**	**Antecedent**							**Consequent**
1	if	ENSG00000129480.11	&	ENSG00000185274.10	&	ENSG00000178163.6	then	Early Stage
2	if	ENSG00000129480.11	&	ENSG00000178163.6	&	--------------------------	then	Early Stage
3	if	ENSG00000129480.11	&	ENSG00000228800.1	&	ENSG00000178163.6	then	Early Stage
4	if	ENSG00000269043.1	&	ENSG00000060339.12	&	ENSG00000178163.6	then	Early Stage
5	if	ENSG00000178163.6	&	ENSG00000060339.12	&	--------------------------	then	Early Stage
6	if	ENSG00000256196.1	&	ENSG00000178163.6	&	ENSG00000060339.12	then	Early Stage
7	if	ENSG00000178163.6	&	ENSG00000060339.12	&	ENSG00000233822.4	then	Early Stage
8	if	ENSG00000228800.1	&	ENSG00000060339.12	&	ENSG00000178163.6	then	Early Stage
9	if	ENSG00000129480.11	&	ENSG00000178163.6	&	ENSG00000233822.4	then	Early Stage
10	if	ENSG00000231475.3	&	ENSG00000178163.6	&	ENSG00000060339.12	then	Early Stage
11	if	ENSG00000129480.11	&	ENSG00000178163.6	&	ENSG00000256196.1	then	Early Stage
12	if	ENSG00000129480.11	&	ENSG00000269043.1	&	ENSG00000178163.6	then	Early Stage
13	if	ENSG00000129480.11	&	ENSG00000231475.3	&	ENSG00000178163.6	then	Early Stage
14	if	ENSG00000129480.11	&	ENSG00000144057.14	&	ENSG00000178163.6	then	Early Stage
15	if	ENSG00000129480.11	&	ENSG00000170011.12	&	ENSG00000178163.6	then	Early Stage
16	if	ENSG00000129480.11	&	ENSG00000178163.6	&	ENSG00000181171.5	then	Early Stage
17	if	ENSG00000144057.14	&	ENSG00000178163.6	&	ENSG00000060339.12	then	Early Stage
18	if	ENSG00000256590.2	&	ENSG00000178163.6	&	ENSG00000060339.12	then	Early Stage
19	if	ENSG00000129480.11	&	ENSG00000253120.1	&	ENSG00000178163.6	then	Early Stage
20	if	ENSG00000170011.12	&	ENSG00000060339.12	&	ENSG00000178163.6	then	Early Stage
**Twenty of the top late-stage rules**								
**No.**	**Antecedent**							**Consequent**
1	if	ENSG00000254463.1	&	ENSG00000233191.1	&	ENSG00000184617.10	then	Late Stage
2	if	ENSG00000113739.9	&	ENSG00000233191.1	&	ENSG00000233235.1	then	Late Stage
3	if	ENSG00000233191.1	&	ENSG00000274698.1	&	ENSG00000113739.9	then	Late Stage
4	if	ENSG00000262038.1	&	ENSG00000233191.1	&	ENSG00000113739.9	then	Late Stage
5	if	ENSG00000159200.16	&	ENSG00000184617.10	&	ENSG00000233191.1	then	Late Stage
6	if	ENSG00000241294.1	&	ENSG00000224287.2	&	ENSG00000272963.1	then	Late Stage
7	if	ENSG00000272963.1	&	ENSG00000233191.1	&	ENSG00000113739.9	then	Late Stage
8	if	ENSG00000277965.1	&	ENSG00000184617.10	&	ENSG00000233191.1	then	Late Stage
9	if	ENSG00000159200.16	&	ENSG00000233191.1	&	ENSG00000113739.9	then	Late Stage
10	if	ENSG00000254463.1	&	ENSG00000233191.1	&	ENSG00000154217.13	then	Late Stage
11	if	ENSG00000254463.1	&	ENSG00000233191.1	&	ENSG00000274698.1	then	Late Stage
12	if	ENSG00000224287.2	&	ENSG00000260498.4	&	ENSG00000272963.1	then	Late Stage
13	if	ENSG00000262038.1	&	ENSG00000233191.1	&	ENSG00000274698.1	then	Late Stage
14	if	ENSG00000184617.10	&	ENSG00000233191.1	&	ENSG00000233235.1	then	Late Stage
15	if	ENSG00000159200.16	&	ENSG00000262038.1	&	ENSG00000233191.1	then	Late Stage
16	if	ENSG00000262038.1	&	ENSG00000233191.1	&	ENSG00000154217.13	then	Late Stage
17	if	ENSG00000184617.10	&	ENSG00000260498.4	&	ENSG00000233191.1	then	Late Stage
18	if	ENSG00000277965.1	&	ENSG00000233191.1	&	ENSG00000113739.9	then	Late Stage
19	if	ENSG00000233191.1	&	ENSG00000154217.13	&	ENSG00000113739.9	then	Late Stage
20	if	ENSG00000233191.1	&	ENSG00000184617.10	&	ENSG00000272963.1	then	Late Stage

**Fig 2:**
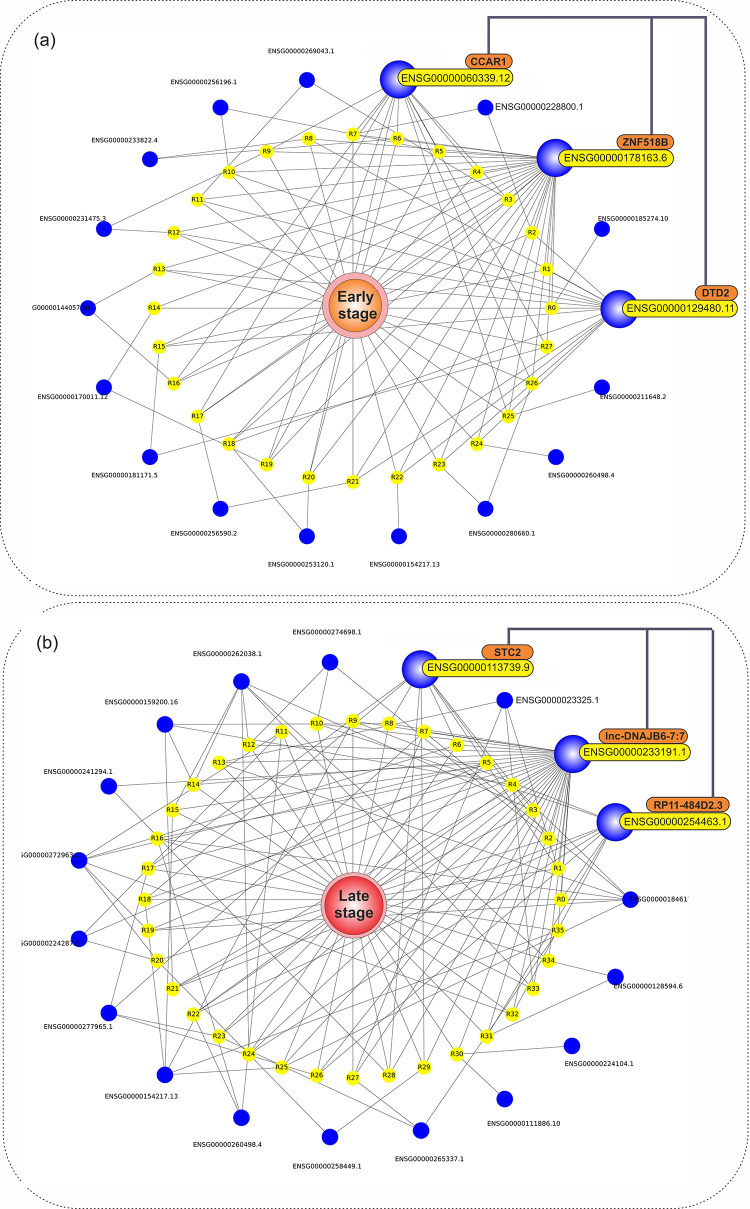
a) Graph network illustration of early-stage related association rules (having lift value > 1.145), in which primary-stage phenotype, its rules, and related mRNAs are shown in orange, yellow, and blue colors, respectively. b) Graph network illustration of late-stage related association rules (having lift value > 1.1), in which the late-stage phenotype, its rules, and related mRNAs are shown in red, yellow, and blue colors, respectively.

Moreover, we have illustrated features-phenotype relations based on extracted association rules in Fig 2 using a graph network representation. Fig 2(A) shows that early-stage phenotype, based on early-stage association rules, has a high dependency on ENSG000001781163.6, ENSG00000129480.11, and ENSG00000060339.12 mRNAs. Looking at Fig 2(B), it is obvious that late-stage phenotype depends on ENSG00000233191.1, ENSG00000254463.1, and ENSG00000224287.2 mRNAs, according to late-stage association rules.

More in-depth biological coverage of these findings is available in the discussion section of the study.

**Fig 3:**
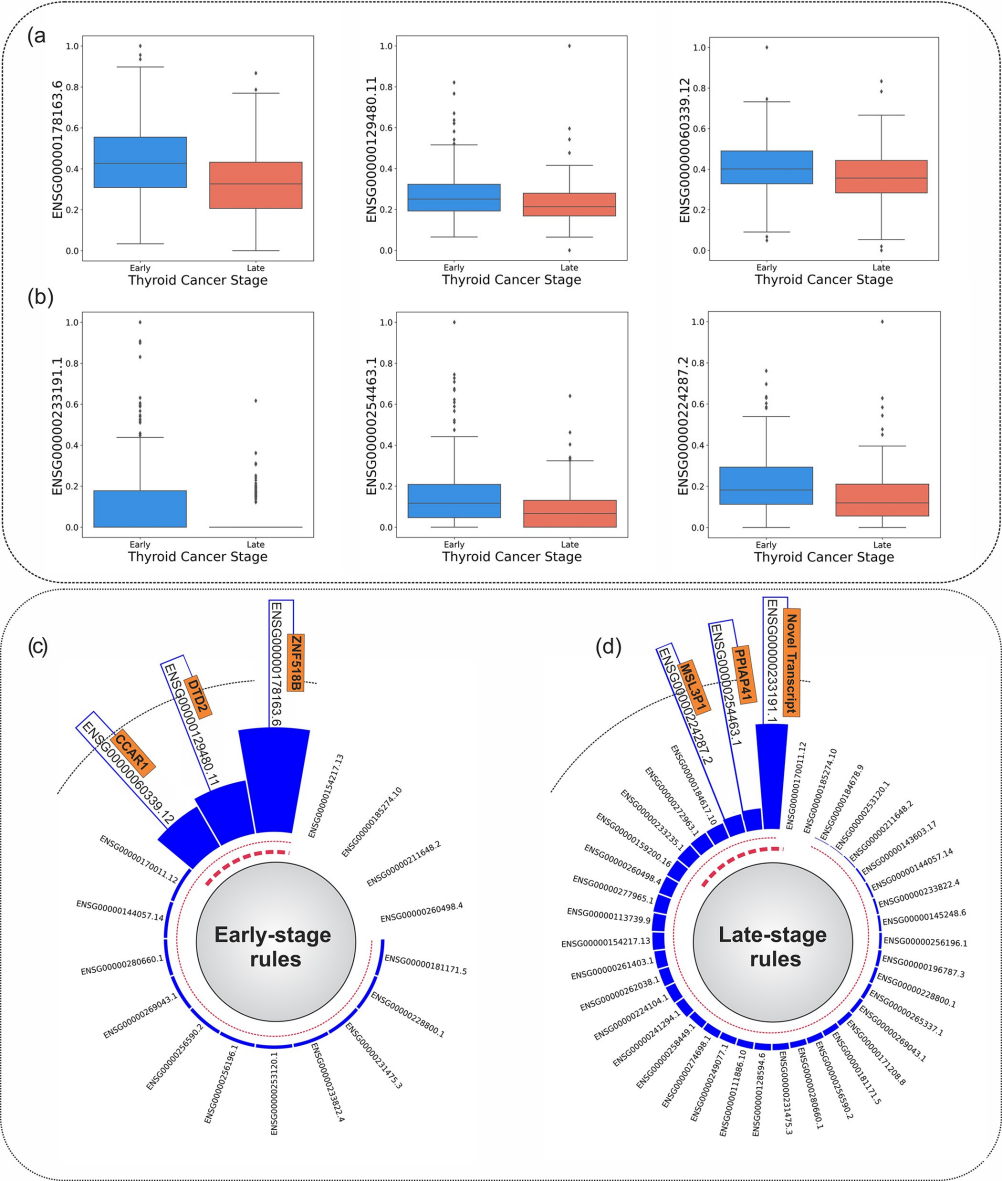
a) Box plot of the selected mRNAs for the early-stage group. b) Box plot of selected mRNAs for the late-stage group. mRNA values have been normalized into the [0 1] range. c) Ring bar plot of early-stage rules based on the count frequency of the top 18 mRNAs. d) Ring bar plot of late-stage rules based on the count frequency of top 39 mRNAs.

Fig 3(A) and 3(B) put the box plots of three significant mRNAs on show for both early and late-stage groups, respectively. A remarkable difference can be noticed through the comparison of medians, interquartile ranges, and whiskers in box plots of significant mRNAs between the two stages of PTC tumors. The next plots, namely Fig 3(C) and 3(D), exemplify the count frequency of top mRNAs in ring bar plots for early-stage and late-stage rules.

**Fig 4:**
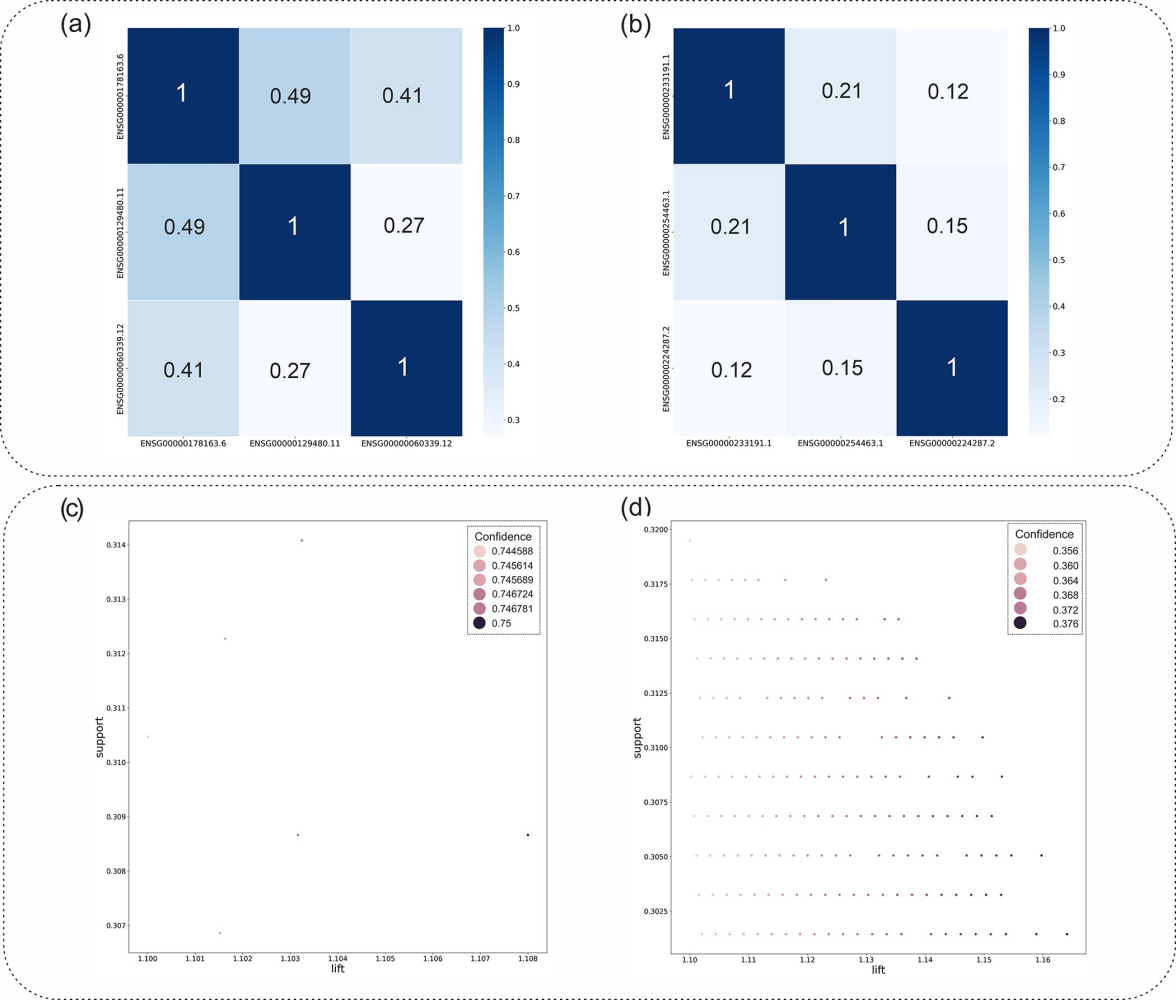
a) The heatmap plot showing the Spearman Correlation for the three top mRNAs of the early-stage rules. b) The heatmap plot showing the Spearman Correlation for the three top mRNAs of the late-stage rules. c) Strength distribution of early-stage association rules based on their support, lift, and confidence scores. d) Strength distribution of late-stage association rules based on their support, lift, and confidence scores.

Fig 4(A) and 4(B) present the Spearman Correlation of the three top mRNAs of the early-stage and late-stage in the form of a heatmap plot, respectively. Moreover, the strength distribution of the early-stage and late-stage association rules has been displayed in the Fig 4(C) and 4(D), based on their support, lift, and confidence scores.

**Fig 5:**
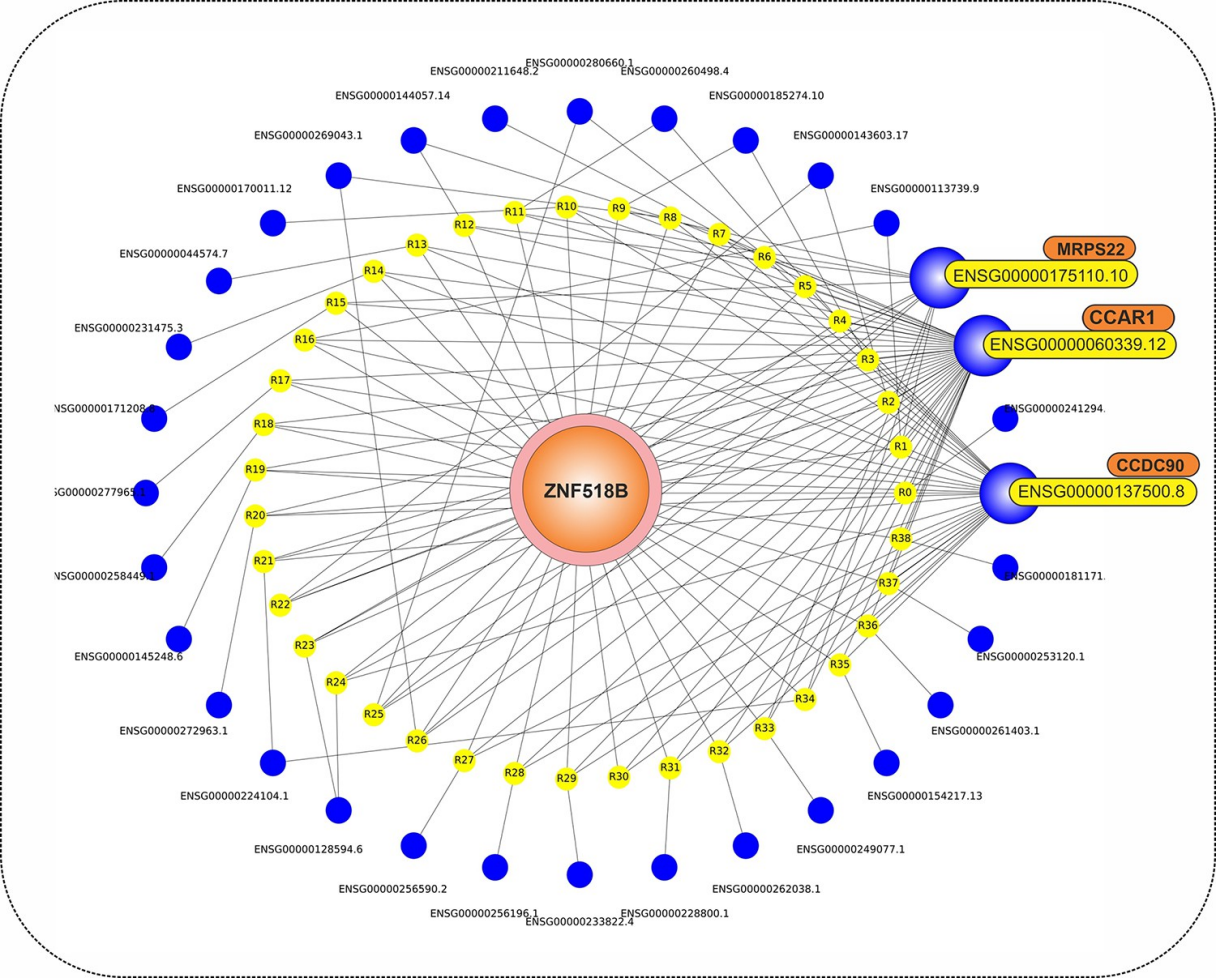
Graph network of ZNF518B (with lift score > 1.23) and its related association rules with other mRNAs. ZNF518B, its rules, and related mRNAs are represented by orange, yellow, and blue colors, respectively.

The ENSG00000178163.6 (ZNF518B) was the most frequent item set with 28 repeat counts in early-stage association rules. As a result, we opted for a closer investigation of this mRNA and its relation with other features using the other association rules. Fig 5 manifests those relations through a graph network in that regard. Additionally, more in-depth biological coverage of these findings is also available in the discussion section of the study. As it is obvious in Fig 5, ZNF518B, the most frequent mRNA in the early-stage association rules, has a high dependency on ENSG0000060339.12, ENSG00000175110.10, and ENSG00000137500.8.

## Discussion

This study aims to identify transcript-based biomarkers capable of predicting the tumor stage of papillary thyroid cancer and differentiating patients with early-stage from late-stage cancer. The diagnosis of PTC at an early stage assists in the management of the disease and the application of acceptable therapy at a proper time, which ultimately would improve the patients’ outcome.

The first identified mRNA that is involved at an early stage of PTC was ZNF518B. Human *ZNF518B* gene is located on chromosome 4 and has five exons and five splicing variants [31], among which, isoforms 1 and 2 are deemed to be the major ones, and the remaining isoforms are considered as non-protein coding. The canonical isoform 1 encodes a protein 1074 residues long, while isoform 2 emerges from the skipping of exons 2 and 3 and a premature transcription termination of exon 4. It includes an open reading frame, which may putatively form a truncated form of the N-terminus of the protein that competes with isoform 1.

The isoform 1 also encodes a zinc-finger protein, a putative transcriptional factor, that interacts with histone methyltransferases (EZH2 and G9A) and activates them [32], consequently, silencing their target genes. Deregulation of G9A has been implicated in several types of cancer [33,34]. Moreover, elevated levels of EZH2 were associated with growth, metastasis, and tumor stage in PTC [35,36]. The isoform 1 of ZNF518B positively modulates histone H3 lysine 9 (H3K9) methylation. The isoform2/isoform1 ratio has been negatively associated with the relapsing of colorectal cancer (CRC) [37]. In tissues and all datasets, the expression of ZNF518B reduces with age and it is a mediator of anti-aging-associated genes [38].

Overall, the interaction of ZNF518B with enzymes can introduce epigenetic alterations, affecting the activity of many genes [37]. The ZNF518B contributes to cell migration and invasiveness, probably through type I collagen fibers [39] and inducing EMT in CRC [40]. It has been proposed that overexpression of ZNF518B might have a role in tumor cell dissemination [41]. Acetylation of histones is involved in the regulation of ZNF518B transcription and histone deacetylases inhibition elevates the expression of ZNF518B [39]. These findings state a potential connection between ZNF518B and cancer. Nevertheless, apart from the aforementioned studies, a few reports are also available in the literature regarding the role of ZNF518B [39,41]. Thus, it appears that ZNF518B could be an invasiveness prognosis target for the development of epigenetic drugs in PTC. As a recognized transcriptional factor, the identification of the possible targets of ZNF518B remains to be determined.

D-aminoacyl-tRNA deacylase (2DTD2), the second identified early-stage marker of PTC, is a checkpoint factor in the translational machinery that prevents chiral errors; it removes the tRNAs-D-amino acids and does not allow D-type amino acids to form proteins. D-amino acids have been long considered to be nonfunctional. Currently, it has been reported that D-amino acids have essential roles in different physiological (regulating gut barrier function and innate immunity) and pathophysiological processes including age-related diseases, schizophrenia, and cancer [42]. The functional role of this enzyme has not yet been studied in the context of cancer; however, our findings suggest that it could be involved in the pathogenesis of early-stage PTC.

Cell cycle and apoptosis regulator 1 (CCAR1/CARP-1), the 3^rd^ identified mRNA in this study, is a biphasic controller of apoptosis and cell growth in cancer. The CCAR1 exerts cell-growth inhibitory and apoptosis-promoting properties through the inhibition of epidermal growth factor receptors [43]. The CCAR1 serves as a cofactor of steroid/thyroid nuclear receptors and p53 in various cancer cells [44] and is required for estrogen-induced gene expression and the estrogen-dependent growth of human breast cancer cells [43]. The CCAR1 is considered as a component of Wnt/β-catenin signaling and is involved in transcriptional regulation by β–catenin [43]. Elevated levels of CCAR1 protein expression were detected in hepatocellular carcinoma (HCC) cases, which might attribute to microvascular invasion, intrahepatic metastasis, higher disease stage, and early recurrence. CCAR1 could predict recurrence-free survival (RFS) in HCC patients following curative hepatectomy and has a critical role as a prognostic marker at early-stage HCC [44]. RNAi-mediated CCAR1 suppression leads to cell growth inhibition, elevated apoptosis, and reduced migration and invasion in gastric cancer [43]. Moreover, the knockdown of long non‑coding RNA (lncRNA) RP11‑284F21.9 successfully inhibits lung tumor growth by downregulation of CCAR1 [45]. It has been shown that circRIMS1 might enhance tumor growth, migration, as well as invasion through the miR-433-3p/CCAR1 regulatory axis [46]. Collectively, evidence indicates that CARP-1/CCAR1 regulates signaling ranging from co-activation of physiological responses to steroids, cell homeostasis, and differentiation, to the chemotherapy-mediated apoptosis signaling [47]. The functional role of CCAR1 in PTC has not yet been studied; it may serve as a potential biomarker and therapeutic target in the early stage of PTC.

In our study, association rule mining identified lnc-DNAJB6-7, PPIAP41 (peptidylprolyl isomerase A pseudogene 41), and MSL3P1 (MSL complex subunit 3 pseudogene 1) genes to be involved at the late stage of PTC. The roles of these genes have not been investigated for PTC in the literature. However, downregulated lnc-DNAJB6-3:1, a long non-coding RNA, was identified as a marker for cartilage endplate degeneration [48]. Moreover, MSL3P1 was among pseudogene transcripts that were deregulated in oropharyngeal cancer [37] and its upregulation is deemed to be a non-invasive biomarker of renal cell carcinoma [36]. The functional roles of these transcripts need to be explored in the pathogenesis of PTC. Table 7 shows mRNAs that were selected using association rule mining analysis, for both early and late stages of PTC. In the present study, we did not evaluate the levels of identified RNAs. It is suggested to assess the expression levels of these RNAs in liquid or tissue biopsy samp les and study their prognostic and diagnostic values in future works.

**Table 7:** Selected mRNAs based on association rule mining analysis, for early and late stages of PTC.

Thyroid Cancer				
**Early-stage**				
	mRNA ID		Full name	Repeat counts
1	ENSG00000178163.6	ZNF518B	Zinc finger protein 518B	28
2	ENSG00000129480.11	DTD2	D-aminoacyl-tRNA deacylase 2	15
3	ENSG00000060339.12	CCAR1	Cell division cycle and apoptosis regulator 1	13
**Late-stage**				
1	ENSG00000233191.1	lnc-DNAJB6-7:7	---	463
2	ENSG00000254463.1	RP11-484D2.3	Peptidylprolyl isomerase A pseudogene 41	104
3	ENSG00000224287.2	MSL3P1	MSL complex subunit 3 pseudogene 1	94

## Conclusion

Recognition of main actor mRNAs involved in PTC and distinguishing between the early and late stages of this cancer are crucial for decoding molecular mechanisms. This could help to identify better therapeutic directions and possible approaches for effective drug development, since targeted molecular therapy with proper timing at an early stage could drastically improve the outcome, and result in lower mortality rates in PTC. Through the present study, we employed an AI-based framework to identify mRNA targets for PTC disease. The proposed approach picked out top three mRNAs for the early stage (e.g., ZNF518B, DTD2, CCAR1), and the late-stage (e.g., lnc-DNAJB6-7:7, RP11-484D2.3, MSL3P1) of PTC. This research also introduces mRNA patterns and association rules which can be helpful in establishing a likely clear picture of significant main actor candidate genes, in both the early and late-stages of PTC. In this study, we did not examine the expression levels of identified RNAs. Future research should analyze these RNAs in liquid or tissue biopsy samples to determine their prognostic and diagnostic values.

## Supporting information

S1 File The Supplementary part comprehensively explains the technical details of each stage in the methodology. (DOCX)
