# Supplementary material for: Uncovering key molecular mechanisms in the early and late-stage of papillary thyroid carcinoma using association rule mining algorithm
Source: PLoS One. 2023 Nov 2;18(11):e0293335. doi: 10.1371/journal.pone.0293335 (PMC10621943; doi:10.1371/journal.pone.0293335)
Supplement: S1 File — (DOCX) [file pone.0293335.s001.docx]

Uncovering Key Molecular Mechanisms in the Early and Late-stage of Papillary Thyroid Carcinoma Using Association Rule Mining Algorithm

Seyed Mahdi Hosseiniyan Khatibi, Ph.D.^1,2,3 †^, Sepideh Zununi Vahed, Ph.D. ^2 †^, Hamed Homaei Rad, Msc^3 †^, Manijeh Emdadi, Ph.D.^4^, Zahra Akbarpour, Msc^3^, Mohammad Teshnehlab, Ph.D.^5^, Saeed Pirmoradi, Ph.D. ^1^ *, Effat Alizadeh, Ph.D. ^6, 7^ *

**Affiliations/institutions**

^1^ Clinical Research Development Unit of Tabriz Valiasr Hospital, Tabriz University of Medical Sciences, Tabriz, Iran

^2^ Kidney Research Center, Tabriz University of Medical Sciences, Tabriz, Iran.

^3^ Rahat Breath and Sleep Research Center, Tabriz University of Medical Science, Tabriz, Iran.

^4^ Department of Computer Engineering, Abadan Branch, Islamic Azad University, Abadan, Iran.

^5^ Department of Electric and Computer Engineering, K.N. Toosi University of Technology, Tehran, Iran.

^6^ Drug Applied Research Center, Tabriz University of Medical Sciences, Tabriz, Iran.

^7^ Department of Medical Biotechnology, Faculty of Advanced Medical Sciences, Tabriz University of Medical Sciences, Tabriz, Iran.

***Corresponding authors:**

Effat Alizadeh

Drug Applied Research Center, Tabriz University of Medical Sciences, Tabriz, Iran.

Email: [e.alizadeh.2010@gmail.com](mailto:e.alizadeh.2010@gmail.com)

Saeed Pirmoradi

Clinical Research Development Unit of Tabriz Valiasr Hospital, Tabriz University of Medical Sciences, Tabriz, Iran

Email: [said.pirmoradi@gmail.com](mailto:said.pirmoradi@gmail.com)

† These authors contributed equally

**Supplementary Method**

**Normalization**

Z-score and min-max normalization formulas are presented in Eqs. 1 and 2. Z-Score uses µ and σ as the mean and standard deviation values of $x$; On the other hand, min-max is calculated using minimum and maximum values of $x$. Changing and converting feature columns of data into normal distribution can be done by applying Z-score. However, the min-max method does not change data distribution and only scales values to [0 1] range.

$y= \frac{x-\mu}{\sigma}$ (1)

$y= \frac{1}{Max-Min}(x-Min)$ (2)

**Nested Cross Validation**

Supervised machine learning and data mining rely on feature selection and classification as their main components. The quality of the classifier is dependent on the quality of selected features. The classifier’s performance is evaluated through its scores on the test data, which is not used for training and validation of the model. Combining too many irrelevant features may lead to low generalization in testing data and high variance error estimation (overfitting) in the training process. In contrast, a lack of significant features may lead to high bias error estimation (underfitting). In this regard, the precise error estimation method plays a crucial role in classification and feature selection procedures.

Cross-Validation (CV) is a fundamental technique used to estimate the accuracy of a classifier in a given dataset by splitting data into training and testing sets. Machine learning researchers have introduced various versions of CV to be applied in feature selection and classifier parameters tuning, including leave-one-out CV, repeated double CV, and nested CV. The nested CV is a reliable way to estimate the accuracy/error of the classifier [1]. In nCV, the data is split into k outer folds, during which the outer fold is held out for testing, and the remaining k-1 folds are merged and split into inner folds for inner training and validation. Folds of outer folds, along with inner training and validation folds, are used for feature selection and model parameters tuning. Finally, general classifier accuracy/error is estimated based on testing scores on outer folds.

**t-test**

The t-test is employed as a statistical test in order to compare the mean of two groups. It is widely used in medical data domain as a parametric statistical hypothesis. Parametric methods make some assumptions about the distribution of variables and parameters of the distribution. Normal distribution of data, equal variance, and independence of samples are the principal assumptions in t-test.

In t-test method [2], t-statistic, based on the Eq. 3, is calculated.

$t= \frac{\left( \bar{x}_{1}-\bar{x}_{2} \right)-(\mu_{1}-\mu_{2})}{\sqrt{\frac{S_{1}^{2}}{n_{1}}+\frac{S_{2}^{2}}{n_{2}}}}$ (3)

In Eq. 4, $\bar{x}_{i}$, $S_{i}^{2}$, $n_{i}$, and $\mu_{i}$ are sample mean, sample variance, number of samples, and population mean, respectively. Eq. 4 converts to Eq. 4 by considering $\mu_{1}-\mu_{2}=0$ based on null hypothesis.

$t= \frac{\left( \bar{x}_{1}-\bar{x}_{2} \right)}{\sqrt{\frac{S_{1}^{2}}{n_{1}}+\frac{S_{2}^{2}}{n_{2}}}}$ (4)

**Non-Dominated Sorting Genetic Algorithm II (NSGAII)**

Multi-objective optimization is an important factor in the multi-criteria decision-making process. It has been employed in many research areas, including engineering, economics, and medicine, in which finding the optimal solution is needed for multi-objective difficulties. Trade-offs between two or more conflicting objectives are the fundamental concept in multi-objective problems, which various algorithms have been designed to solve. Meta-heuristic algorithms are robust procedures in optimization problems with incomplete data or limited calculation ability. Meta-heuristics do not find a globally optimal solution on some problems versus other optimization algorithms and iterative methods [3].

NSGA-II is the well-known meta-heuristic optimization method for multi-objective optimization [4]. Its full name is "Non-dominated Sorting Genetic Algorithm II" and is the updated version of NSGA. It finds solutions for non-convex, non-smooth single and multi-objective optimization problems. It utilizes a new mating mechanism based on the crowding distance and considering constraints using an adapted explanation of dominance without applying penalty functions. The whole process of NSGAII is illustrated in Table 1.

**Table 1.** Pseudocode of NSGAII [5]

| **Algorithm:** Non-dominated Sorting Genetic Algorithm (NSGA-II) |
| --- |
| 1. Create an initial population P_0_  2. Create an offspring population Q_0_  3. t = 0  4. **while** stopping criteria not reached **do:**  5. R_t_ = P_t_ ∪ Q_t_  6. F = fast-non-dominated-sort (R_t_)  7. P_t+1_ = ∅ and i = 1  8. **while** \|P_t+1_\| + \|F_i_ \| ≤ N **do:**  9. Apply crowding-distance-assignment (F_i_)  10. P_t+1_ = P_t+1_ ∪ F_i_  11. i = i + 1  **end**  12. Sort (F_i_ , ≺ n)  13. P_t+1_ = P_t+1_ ∪ F_i_ [N − \|P_t+1_\|]  14. Q_t+1_ = create_new_pop (P_t+1_)  15. t = t + 1  **end** |

**Classification**

Classifier models apply well-known machine learning algorithms for classification tasks. In this study, we used Support Vector Machine (SVM), Naive Bayes (NB), K-Nearest Neighbor (KNN), Random Forest (RF), as classic classifiers, and Deep Self-Organizing Auto-Encoder (SOAE) [6] to assess the differentiation power of selected features.

We evaluated the predictive performance of the classifiers using the following metrics (Eqs. 5-8), including accuracy, F1-score, Matthews Correlation Coefficient (MCC), the Area Under the ROC Curve (AUC), sensitivity (Sn), and specificity (Sp).

$Accuracy= \frac{TP+TN}{TP+TN+FP+FN}\times100$ (5)

$F_{1}=\frac{TP}{TP+\frac{1}{2}(FP+FN)}$ (6)

$sensitivity= \frac{TP}{TP+FN}\times100$ (7)

$specificity=\frac{TN}{TN+FP}\times100$ (8)

Where TP, TN, FP, and FN are True Positive, True Negative, False Positive, and False Negative, respectively.

**Association Rule Mining**

Association rule mining is a powerful data mining tool that presents the hidden association in the form of rules by discovering associated frequently co-occur items in the dataset. Market basket analysis [7] and bioinformatics [8] are two main areas that apply association rule mining to extract the significant association in marketing and genomic data, respectively. The interpretation of gene expression data (mRNA), annotations, detection of protein interaction, and biomolecular localization prediction are some applications of association rule mining in bioinformatics [8].

Association rule mining has two main steps, frequent itemset mining, and association rule generation. Frequent itemset mining (FIM) extracts frequently co-occur sets of items (i.e., frequent itemsets). If itemset support value is more than the minimum support threshold, itemset is called a frequent itemset. Next, the association rule generation step creates the rules from the discovered Frequent Itemsets (FIs). If support/confidence/lift value of the rule is no less than the minimum support/confidence/lift threshold value, the generated rule is called the association rule. These thresholds are user-defined parameters.

The association rule mining is an NP-hard problem, in which finding the results are challenging in a reasonable time. Introducing the Apriori algorithm addressed the computational problem in most regular-sized data [9]. Since then, many types of research have been done to develop new algorithms such as FP-Growth [10] and Eclat [11]. These algorithms improved the scalability of the Apriori algorithm. However, the relative computational cost of the FIM stage during association rule mining for high-dimensional data and big data is still a challenging issue.

The above section mentions some principal ideas and terms used in association rule mining. In the following, we describe and formalize these basic concepts of frequent itemset and association rule. The related theories are available in [12] with more details. Let $I=\{i_{1}, i_{2}, \ldots,i_{d}, y\}$ is a set of items, $D=\{d_{1}, d_{2}, \ldots, d_{n}\}$ is a dataset of n instances, $F=\{f_{1}, f_{2}, \ldots, f_{m}\}$ is the features space with m features, and $Y=\{0, 1\}$ is the user-defined phenotype. The $d_{i}$ can be presented as a tuple $(X_{i}, y_{i})$, where $X_{i}\in f_{1}\times f_{2}\times\ldots\times f_{m}$ and $y_{i}\in Y$.

*Definition 1.* (Length of an itemset)

Let $X$ be an itemset, which has K-distinct items, the length of the $X$ is defined as $|X|=K$.

*Definition 2.* (Support count and support of an itemset)

The total number of samples including $X$ itemset is defined support count of an itemset $X$. Also, support of an item set $X$ is the ratio of support count to the total number of samples.

*Definition 3.* (Frequent itemset)

An itemset $X$ is called a frequent itemset if and only if its support is no less than the minimum support, which is the user-defined threshold.

*Definition 4.* (Association rule)

An association rule is defined as a form of $A\to C$, where $A$ and $C$ are itemsets and $A\cup C=\varphi$, $A\subset X, C\subset X$. In the $A\to C$, $A$ and $C$ are called the Antecedent and Consequent, respectively. Also, $A\to C$ displays the association that if all items in Antecedent occur, then all items in Consequent co-occur. The generated association rules are filtered out based on the user-defined threshold, such as support, confidence, and lift.

*Definition 5.* (Support of rule)

The support of rule $A\to C$ is the percentage of samples in D (as shown in Eq. 10). This measure presents the usefulness of the rule.

$Support\left( A\to C \right)= \frac{support(A\cup C)}{n}$ (10)

*Definition 6.* (Confidence of rule)

The confidence of rule $A\to C$ is the percentage value that displays how frequently $C$ occurs among all the examples containing $A$ (as shown in Eq. 11). This measure shows the certitude of the rule.

$Confidence\left( A\to C \right)=P\left( C | A \right)= \frac{support(A\cup C)}{support(A)}$ (11)

*Definition 7.* (Lift of rule)

The Lift of rule $A\to C$ determines the dependency between the occurrence of itemsets $A$ and $C$. When the Lift value is more (less) than one, the occurrence of $A$ is positively (negatively) correlated with the occurrence of $C$. If the Lift value is equal to one, then $A$ and $C$ are independent. The Lift value is shown in Eq. 12.

$Lift\left( A\to C \right)= \frac{P(A\cup C)}{P\left( A \right)P(C)}$ (12)

In this study, we apply FP-Growth approach for association rule mining. Also, the pseudo-codes of two stages are available in Tables 2 and 3.

**Table 2.** Pseudocode of frequent itemset generation step in FP-Growth algorithm [10]

| **Algorithm:** Frequent itemset generation in FP-Growth algorithm |
| --- |
| **Input:** A database DB, represented by FP-tree constructed, and a minimum support threshold ξ.  **output:** The complete set of frequent patterns  **Method:** FP-growth (Tree, α)  (1) **if** Tree contains a single prefix path, **then**: // Mining single prefix-path FP-tree  (2) let P be the single prefix-path part of Tree;  (3) let Q be the multipath part with the top branching node replaced by a null root;  (4) **for** each combination (denoted as β) of the nodes in the path P **do**:  (5) generate pattern $\beta\cup\alpha$ with support = minimum support of nodes in β;  (6) let freq_pattern_set(P) be the set of patterns so generated;  (7) **end for**  (8) **else** let Q be Tree:  (9) **for** each item ai in Q **do**: // Mining multipath FP-tree  (10) generate pattern $\beta= a_{i} \cup\alpha$ with support = $a_{i}$.support;  (11) construct β’s conditional pattern-base and then β’s conditional FP-tree Treeβ;  (12) **if** Treeβ = ∅ **then**:  (13) call FP-growth (Treeβ, β);  (14) let freq_pattern_set(Q) be the set of patterns so generated;  (15) **end if**  (16) **end for**  (17) **end if**  (18) return (freq_pattern_set(P) ∪ freq_pattern_set(Q) ∪ (freq_pattern_set(P) × freq_pattern_set(Q))) |

**Table 3.** Pseudocode of rules generation step in FP-Growth algorithm [13]

| **Algorithm:** Rules generation in FP-Growth algorithm |
| --- |
| **Method:**  (1) **For** each frequent itemset k-itemset F_k_, k≥2 **do:**  (2) $H_{1}=\{i\vert i\in F_{k}\}$; // 1-item consequents of the rule.  (3) call rules_generator (F_k_, H_1_);  (4) **End for**  Procedure rules_generator (F_k_, H_m_)  (1) $k=\vert F_{k}\vert$; // size of frequent itemset  (2) $m=\vert H_{m}\vert$; // size of rule consequent  (3) **if** k>m+1 **then:**  (4) $H_{m}=m+1-item consequents generated from H_{m}$;  (5) **For** each $h_{m+1}\in H_{m+1}$ **do:**  (6) $Confidance= \frac{Support(F_{k})}{Support(F_{k}-H_{m+1})}$;  (7) **if** Confidence ≥ min_confidence **then:**  (8) output: the rule (F_k_ – h_m+1_) → h_m+1_;  (9) **Else:**  (10) delete h_m+1_ from H_m+1_;  (11) **End if**  (12) **End for**  (13) call ap-genrules(F_k_, H_m+1_)  (14) **End if** |

**References**

1. Parvandeh, S., et al., *Consensus features nested cross-validation.* Bioinformatics, 2020. **36**(10): p. 3093-3098.

2. Kim, T.K., *T test as a parametric statistic.* Korean journal of anesthesiology, 2015. **68**(6): p. 540-546.

3. Hojjati, A., et al., *Application and comparison of NSGA-II and MOPSO in multi-objective optimization of water resources systems.* J Hydrol Hydromech, 2018. **66**(3): p. 323-329.

4. Deb, K., et al., *A fast and elitist multiobjective genetic algorithm: NSGA-II.* IEEE Transactions on Evolutionary Computation, 2002. **6**(2): p. 182-197.

5. Soui, M., et al., *NSGA-II as feature selection technique and AdaBoost classifier for COVID-19 prediction using patient’s symptoms.* Nonlinear Dynamics, 2021: p. 1-23.

6. Pirmoradi, S., et al., *A Self-organizing Deep Auto-Encoder approach for Classification of Complex Diseases using SNP Genomics Data.* Applied Soft Computing, 2020: p. 106718.

7. Kaur, M. and S. Kang, *Market Basket Analysis: Identify the changing trends of market data using association rule mining.* Procedia computer science, 2016. **85**: p. 78-85.

8. Naulaerts, S., et al., *A primer to frequent itemset mining for bioinformatics.* Briefings in bioinformatics, 2015. **16**(2): p. 216-231.

9. Agrawal, R. and R. Srikant. *Fast algorithms for mining association rules*. in *Proc. 20th int. conf. very large data bases, VLDB*. 1994. Citeseer.

10. Han, J., et al., *Mining frequent patterns without candidate generation: A frequent-pattern tree approach.* Data mining and knowledge discovery, 2004. **8**(1): p. 53-87.

11. Zaki, M.J., *Scalable algorithms for association mining.* IEEE transactions on knowledge and data engineering, 2000. **12**(3): p. 372-390.

12. Li, H. and P.C.Y. Sheu, *A scalable association rule learning heuristic for large datasets.* Journal of Big Data, 2021. **8**(1): p. 86.

13. Xie, J., J. Wu, and Q. Qian. *Feature selection algorithm based on association rules mining method*. in *2009 Eighth IEEE/ACIS International Conference on Computer and Information Science*. 2009. IEEE.
